# Reply letter for ‘important factors affecting red blood cell distribution shouldn’t be ignored’

**DOI:** 10.1080/0886022X.2022.2124174

**Published:** 2022-09-21

**Authors:** Rongqian Hua, Xuefang Liu, Enwu Yuan

**Affiliations:** Department of Clinical Laboratory, The Third Affiliated Hospital of Zhengzhou University, Zhengzhou, China

Dear Editor,

With respect to the recent letter of Zhao and his coworkers [1], commenting on our recent paper published in Renal Failure [2], we appreciated the opportunity to elaborate on our results. The authors mainly talked about four concerns with regard to our study. Firstly, we did not include albumin as a confounding factor in our study. Secondly, we missed out nutritional data, bone marrow function parameters and blood transfusion data in our study. Thirdly, we were suggested to use standard deviation of the red blood cell distribution (SD-RBC) to replace red cell distribution width (RDW). Finally, some problems in our reference list.

Firstly, about albumin mentioned by the author. We have read reference 3 in this letter. The authors showed correlations and linear regressions of baseline RDW with various laboratory data, and their analysis showed that the albumin was negatively correlated with RDW in patients with kidney failure [3]. Albumin is a commonly used index to detect liver function. The decrease of albumin not only represents malnutrition, but also reflects the decline of liver function. Hu et al. [4] showed that increased RDW was associated with worse hospital outcome of liver function. Similarly, our data in Table 1 [2] also showed that kidney failure patients in the high RDW group (≥17.2%) were more likely to have severe liver disease complications. We think that the negative correlation between RDW and albumin is probably connected by changes in liver function. Therefore, in the experimental design, we did not include albumin.

Secondly, about the lack of nutrition data, bone marrow function parameters and blood transfusion data. In the part of materials and methods in our paper, we explained that the RDW value and other laboratory results we selected were the values when the patients were admitted to ICU for the first time. If more than one fifth of the patients lacked a laboratory result, we had to remove this parameter to ensure the accuracy of our analysis results. Unfortunately, some laboratory results like ferritin, CRP were excluded by this reason. However, we listed some other nutrition data like hemoglobin in our Table 1. As for the bone marrow function parameters, we listed the platelet count in our table and used it as an adjustment parameter in the Cox proportional hazards regression model and subgroup analysis. Maybe you missed it while reading our tables.

For the blood transfusion data, we mentioned that we only focused on levels of RDW and other parameters at admission to the ICU in the discussion part of our study. Therefore, in the design of the experiment, we did not pay too much attention to this index, because blood transfusion data only affected the fluctuation of RDW value during the treatment process but did not affect the RDW value at admission.

Thirdly, on the concern of using SD-RBC to replace RDW. Both RDW and SD-RBC are parameters reflecting the heterogeneity of red blood cell volume. The reason why RDW is selected in this paper is that RDW is widely used instead of SD-RBC in most of the previous studies. However, after reading your letter, we will pay more attention to SD-RBC in our further study of kidney failure.

At last, the author found there were some problems with the sequence of references from 14 to 17. After reading these comments, we rechecked our references list immediately. The references 14 and 15 should be 16 and 17, and the references 16 and 17 should be 14 and 15. We apologize that we did not find this problem in the proof stage and we are sorry for the inconvenience caused to readers’ reading and editors’ work.
